# CRTAC1 identified as a promising diagnosis and prognostic biomarker in lung adenocarcinoma

**DOI:** 10.1038/s41598-024-61804-x

**Published:** 2024-05-16

**Authors:** Lin Tan, Han Zhang, Yun Ding, Yangyun Huang, Daqiang Sun

**Affiliations:** 1 Tianjin Medical University Graduate School, Tianjin, China; 2 Qingdao Hospital, University of Health and Rehabilitation Sciences (Qingdao Municipal Hospital), Qingdao, China; 3 Tianjin Chest Hospital, Tianjin University, Tianjin, China; 4 Clinical School of Thoracic, Tianjin Medical University, Tianjin, China

**Keywords:** LUAD, Pyroptosis, Diagnosis, Prognosis, CRTAC1, Cancer, Cell biology, Biomarkers, Oncology

## Abstract

CRTAC1, one of the pyroptosis-related genes, has been identified as a protective factor in certain kinds of cancer, such as gastric adenocarcinoma and bladder cancer. The study aimed to investigate the role of CRTAC1 in lung adenocarcinoma (LUAD). LUAD datasets were obtained from Gene Expression Omnibus (GEO) database and The Cancer Genome Atlas (TCGA), pyroptosis-related genes from GeneCard. Limma package used to find differentially expressed genes (DEGs), least absolute shrinkage and selection operator (LASSO) regression and weighted genes co-expression network analysis (WGCNA) to identify CRTAC1 as hub gene. CRTAC1 expression was confirmed in a real-world cohort using quantitative polymerase chain reaction (qPCR) and Western Blot (WB) analyses. Cellular experiments were conducted to investigate CRTAC1’s potential oncogenic mechanisms. CRTAC1 mRNA expression was significantly lower in LUAD tissues (*p* < 0.05) and showed high accuracy in diagnosing LUAD. Reduced CRTAC1 expression was associated with a poor prognosis. Higher CRTAC1 expression correlated with increased immune cell infiltration. Individuals with high CRTAC1 expression showed increased drug sensitivity. Additionally, qPCR and WB analyses showed that CRTAC1 expression was lower in tumor tissue compared to adjacent normal tissue at both the RNA and protein levels. Upregulation of CRTAC1 significantly inhibited LUAD cell proliferation, invasion, and migration in cellular experiments. CRTAC1 has the potential to serve as a diagnostic and prognostic biomarker in LUAD.

## Introduction

LUAD exhibits a greater incidence rate and susceptibility to lymph node and distant metastasis^1^. Although certain novel diagnostic modalities have shown advantages in the context of LUAD, it is unfortunate that an estimated 70% of patients are diagnosed only after the disease has progressed to an advanced stage^2^. Furthermore, in addition to conventional radiation and chemotherapy, an increasing number of molecularly targeted agents and immune checkpoint inhibitors (ICI) are being utilized to manage LUAD^3–5^. Nevertheless, the clinical outcomes of LUAD treatment remain suboptimal, as evidenced by a 5-year survival rate of below 30%^5^. Thus, the need for new treatment strategies is evident, particularly in light of recent advancements in bioinformation technology. To this end, identifying a novel set of biomarkers capable of forecasting the diagnosis and prognosis is imperative.

Pyroptosis, a recently discovered form of programmed cell death with inflammatory properties, has been identified as a critical factor in tumor progression^6^. Prior investigation has indicated a significant correlation between several pyroptosis-related genes, namely NLRP7, NLRP1, NLRP2, NOD1, and CASP6, and the immune cells infiltration as well as tumor mutation burden. This correlation has facilitated the ability to forecast the overall survival (OS) of individuals with LUAD^7^. Additionally, Song et al.^8^ suggested that pyroptosis-related genes, such as CCR2, GNG10, CASP4, COTL1, DOK1, AQP8, and FCRLB, may possess clinical significance not only in prognostic prediction but also in the effectiveness of chemotherapy and immunotherapy. Consequently, there exist justifications to posit that pyroptosis-related genes may exhibit a strong correlation with the advancement and management of LUAD. However, the current discovery of these genes did not significantly improve the diagnosis and treatment of LUAD. It is necessary to explore the new diagnostic and prognostic biomarker associated with pyroptosis.

CRTAC1, a pyroptosis-related gene, has been shown to be related to the development of tumor. Patients with high CRTAC1 expression levels exhibit a more favorable prognosis in bladder cancer and glioma with respect to those whose expression levels are low^9,10^. In the case of lung cancer, prior research has demonstrated a decrease in CRTAC1 expression in lung adenocarcinoma tissues compared to normal tissues^11^, with elevated levels of CRTAC1 enhancing the chemosensitivity of non-small cell lung cancer (NSCLC) to cisplatin therapy^12^. However, the current understanding of the involvement of CRTAC1 in the pathogenesis of lung cancer remains incomplete. This study further investigates the role of CRTAC1 in the malignant progression of LUAD through a comprehensive bioinformatics analysis, identifying CRTAC1 as a significant biomarker for diagnostic and prognostic purposes. Furthermore, the drug sensitivity analysis revealed that LUAD patients who exhibited elevated CRTAC1 expression demonstrated a greater responsiveness to drug therapy. Moreover, the results obtained from qPCR and WB experiments confirmed that the CRTAC1 expression level in tumor tissues exhibited a significant decrease in comparison to that of normal tissues. Finally, a set of cellular experiments provided further validation for the precision of our analysis and highlighted CRTAC1 as a prospective therapeutic target for LUAD.

## Materials and methods

### Materials and samples

GEO database (http://www.ncbi.nlm.nih) was assessed, and GSE31210 (n = 246) and GSE68465 (n = 462) datasets were recruited. Furthermore, clinical information and mRNA sequencing profiles were gained from TCGA (https://portal.gdc.cancer.gov/). 598 samples were involved in the further analysis. GeneCards, the gene card database (https://www.genecards.org/), was utilized to acquire 792 pyroptosis-related genes for further analysis.

### Differential expression analysis

Data analyses were performed using R statistical language (version 4.2.2). The DEGs were identified through the application of the limma package to the 2 GEO databases, with the cutoff criteria (*p*.adj < 0.05 and |log2FC|> 1). After overlapping DEGs and pyroptosis-related genes utilizing the VennDiagram package, 31 pyroptosis-related DEGs were chosen for subsequent LASSO analysis and LASSO regression model was implemented using the glmnet package. The identified DEGs underwent Gene Ontology (GO) and Kyoto Encyclopedia of Genes and Genomes (KEGG) pathway enrichment analysis through utilization of the clusterProfiler package, with visualization of the results accomplished through employment of the ggplot2 package. A cut-off criterion of *p* < 0.05 was established. Subsequently, a protein–protein interaction (PPI) network of DEGs was established by utilizing the Search Tool for the Retrieval of Interacting Genes (STRING, http://string.embl.de/) database. The ggraph package was utilized to visualize the network, with a confidence score of ≥ 0.4 serving as the designated cut-off criterion.

As WGCNA, an unsupervised method, contributed to identifying the correlation between the potential biomarker and the mechanisms of disease, we employed the WGCNA package to identify the most significant gene co-expression module associated with disease and investigate the hub genes within the module. In WGCNA, Module Membership (MM) refers to the correlation between individual genes and their corresponding modules, whereas Gene Significance (GS) refers to the correlation between individual genes and traits contained within those modules. Therefore, genes with higher |MM|> 0.75and |GS|> 0.5 are designated as hub genes.

### CRTAC1 expression in LUAD and its prognostic and diagnostic value

The mRNA expression of CRTAC1 was analyzed utilizing the TCGA and GEO datasets. In addition, the Human Protein Atlas database (HPA) (http://www.proteinatlas.org/) was employed to analyze the protein expression level of CRTAC1. The patients were stratified into two groups namely low and high CRTAC1 expression groups, based on their CRTAC1 expression levels, utilizing the median expression value as the demarcation point, and the two groups were analyzed for DEGs (*p.adj* < 0.05; |log_2_FC|> 1). To explore potential mechanisms underlying correlation between the CRTAC1 expression level and LUAD, Gene Set Enrichment Analysis (GSEA) was conducted through cluster Profiler package. The connection between CRTAC1 expression level and various clinic pathologic features in lung cancer cohort was also examined utilizing the stats package. Additionally, the diagnostic and prognostic efficacy of CRTAC1 was evaluated through the utilization of the ROC (receiver operating characteristic) curve and Kaplan–Meier curve utilizing the pROC and survival packages. A nomogram was generated utilizing the LASSO regression analysis to forecast the risk of patients using the survival and rms packages. Additionally, to assess the prognostic precision of the nomogram, calibration plots were employed using the survival and rms packages.

### The transcription factor (TF) network and microRNA (miRNA) network

Three transcription factor prediction databases were used to forecast transcription factors targeting CRTAC1: JASPAR (http://jaspar.genereg.net), AnimalTFDB (http://bioinfo.life.hust.edu.cn/AnimalTFDB4/), and GTRD (http://gtrd.biouml.org/). miRNAs targeting CRTAC1 were predicted based on miRDB (http://mirdb.org/), TargetScan (https://www.targetscan.org/), and miRWalk (http://mirwalk.umm.uni-heidelberg.de). The VennDiagram package was utilized to graph the intersection set of findings from three databases. The correlation and survival analyses of TFs were carried out using the ggplot2 and survival packages.

### CRTAC1 mutational analysis, methylation analysis and single-cell analysis

CRTAC1 mutational analysis, methylation analysis and single-cell analysis were performed separately using cBioPortal (http://www.cbioportal.org), Gene Set Cancer Analysis (GSCA; http://bioinfo.life.hust.edu.cn/GSCA/) and PanglaoDB (https://panglaodb.se/).

### The correlation analysis between CRTAC1 and the immune cell infiltration and immune checkpoint genes

GSVA package was employed to compute the enrichment score of 24 distinct kinds of immune cells through the implementation of the single-sample Gene Set Enrichment Analysis (ssGSEA) algorithm. Subsequently, the Spearman correlation test, implemented via the ggplot2 package, was utilized to look for the interaction between immune cell infiltrates and CRTAC1 expression. Additionally, the aforementioned method was also used to assess CRTAC1 and immune checkpoints expression correlations.

### Analysis of drug sensitivity

The Genomics of Drug Sensitivity in Cancer (GDSC; https://www.cancerrxgene.org/) database was the source of the data pertaining to drug sensitivity. The determination of drug sensitivity is on the basis of the half maximal inhibitory concentration (IC50) of the drug using the oncoPredict package.

### The expression validation of CRTAC1 by qPCR and WB

The SYBR^®^ Green (Roche, Basel, Switzerland) was utilized for the quantification of qPCR analysis. The 2-ΔΔCt method was employed to ascertain the relative expression of CRTAC1, with GAPDH serving as an internal reference. The primers utilized in this research were documented in the Supplement Table 1. All data were analyzed by GraphPad Prism version 9.1.0. A *p*-value below 0.05 was deemed as statistically significant. The protein lysates were subjected to separation using a 4–20% precast sodium dodecyl sulfate polyacrylamide gel electrophoresis (SDS-PAGE) system (GenScript, Nanjing, China) and subsequently transferred onto polyvinylidene fluoride (PVDF) membranes (Millipore, Billerica, MA, USA) for analysis. The membranes were subjected to an overnight incubated with rabbit polyclonal anti-CRTAC1 antibody (1:500 dilution, 13,001-1-AP; Proteintech Group, USA) and mouse anti-GAPDH antibody (1:100,000 dilution, 60,004–1-Ig; Proteintech Group, USA) at a temperature of 4 °C. Following the introduction of HRP-conjugated Affinipure Goat Anti-Rabbit IgG(H + L) (1:10,000 dilution; SA00001-2, Proteintech Group, USA) and HRP-conjugated Affinipure Goat Anti-Mouse IgG(H + L) (1:5,000 dilution; SA00001-1, Proteintech Group, USA), an incubation period of 1.5 h was permitted. WB analysis was conducted using Image Lab software.

**Table 1:** The relationship between CRTAC1 expression and clinicopathological parameters in individuals diagnosed with NSCLC.

Characteristics	Low expression of CRTAC1	High expression of CRTAC1	P value
n	520	521	
Pathologic T stage, n (%)			0.001
T1	118 (11.4%)	172 (16.6%)	
T2	307 (29.6%)	279 (26.9%)	
T3	69 (6.6%)	51 (4.9%)	
T4	25 (2.4%)	17 (1.6%)	
Pathologic N stage, n (%)			0.012
N0	319 (31.3%)	351 (34.4%)	
N1&N2&N3	195 (19.1%)	154 (15.1%)	
Pathologic M stage, n (%)			0.466
M0	391 (48.3%)	386 (47.7%)	
M1	14 (1.7%)	18 (2.2%)	
Pathologic stage, n (%)			0.003
Stage I	246 (23.9%)	295 (28.7%)	
Stage II	166 (16.1%)	121 (11.8%)	
Stage III & Stage IV	104 (10.1%)	97 (9.4%)	
Age, n (%)			0.243
< = 65	233 (23%)	215 (21.2%)	
> 65	273 (26.9%)	292 (28.8%)	
Gender, n (%)			0.004
Male	333 (32%)	288 (27.7%)	
Female	187 (18%)	233 (22.4%)	

### Cell line culture

In our research, BEAS-2B normal human lung epithelial cells and LUAD cell lines (A549, H1650, H1975, H1299) were obtained from the Shanghai Institute for Biological Sciences’ Cell Resource Center. These cells were subsequently cultured in RPMI-1640 medium (manufactured by Gibco BRL, USA), supplemented with 10% fetal bovine serum (FBS) sourced from Cell-Box in Hong Kong, and a 1% penicillin–streptomycin solution provided by Biosharp in China. The cells were maintained in a controlled environment with 5% CO_2_, 95% humidity, and a constant temperature of 37 °C.

### Assessment of cell proliferation using the CCK-8 method, colony formation and EdU

CCK-8 commenced by seeding cells at a density of 3 × 10^3^ cells/well in 96-well plates. Following this, each well received a treatment of 10 mL of CCK-8 reagent (A311-01, Vazyme), and the plates were incubated in darkness at 37 °C for a duration of 2 h. To evaluate cell proliferation, the absorbance at 450 nm was measured using a spectrophotometer (A33978, Thermo Fisher Scientific) at various time points: 0, 24, 48, and 72 h. Colony Formation was commenced by inoculating cells at a density of 1 × 10^3^ cells/well in 6-well plates, which were then cultured for a period of 14 days. Subsequently, the cells were washed with PBS and subsequently fixed with 4% paraformaldehyde for a duration of 15 min. Crystal Violet, supplied by Solarbio, China, was employed for the staining process. Cells were seeded at a density of 2 × 10^4^ cells/well in 96-well plates and treated with EdU (Beyotime Biotechnology, Shanghai, China). Following EdU treatment, the cells were incubated until attachment, and subsequently subjected to nuclei staining usi-ng Hoechst 33,342. The resulting cells were then observed under a fluorescence micro-scope.

### Migration and invasion analysis via transwell assays

The migration and invasion capacities of A549 and H1650 cells were assessed using 24-well transwell inserts. The upper chamber was seeded with 1 × 10^5^ cells, and matrigel from BD Biosciences, USA was applied as a coating for invasion assays. However, for migration assays, the chambers remained uncoated. After the migration or invasion process, cells on the lower side of the membrane were fixed and stained with crystal violet obtained from Solarbio, China.

### Statistical analysis

The statistical analyses for the bioinformatics part were performed within the R environment (version 4.2.2), whereas Graphpad, ImageJ and Image Lab (version 6.1, https://www.bio-rad.com/zh-cn/product/image-lab-software?ID=KRE6P5E8Z)were utilized for statistical analysis of basic experimental data. The Chi Square test was employed to examine variables that are categorical in nature. The independent sample T-test or one-way ANOVA was utilized to assess intergroup differences in samples that exhibited a normal distribution. In cases where the samples did not conform to a normal distribution, the Wilcoxon rank-sum test or Kruskal–Wallis test was employed. The Spearman’s correlation test and log-rank survival analysis test were utilized in this study. A *p*-value below 0.05 was deemed as statistically significant, as showed by asterisks (*, **, ***, and ****, corresponding to *p* < 0.05, *p* < 0.01,* p* < 0.001, and *p* < 0.0001, respectively).

### Ethical approval and consent to participate

All studies involving human tissues were conducted in strict accordance with the Declaration of Helsinki and were approved by the Ethics Committee of Tianjin Chest Hospital (protocol code: IRB-SOP-016(F)-001-02). All participants were informed and consent to sample collection, intended research, and publication usage. Written consent was collected according to the ethical regulations of Tianjin Chest Hospital.

## Results

### Differential expression analysis

**Figure 1:**
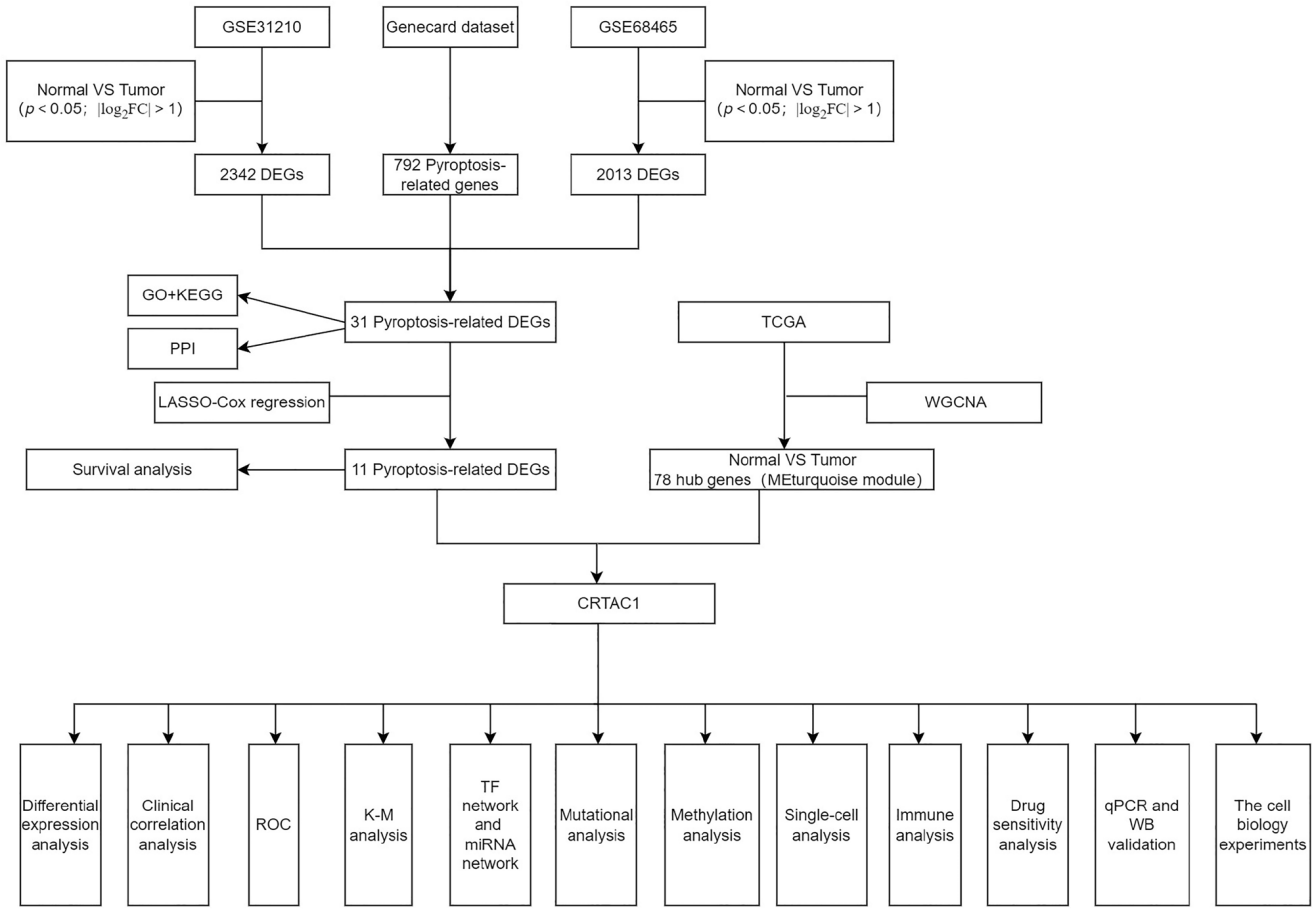
The flowchart of data collection, analysis, and experimental validation.

**Figure 2:**
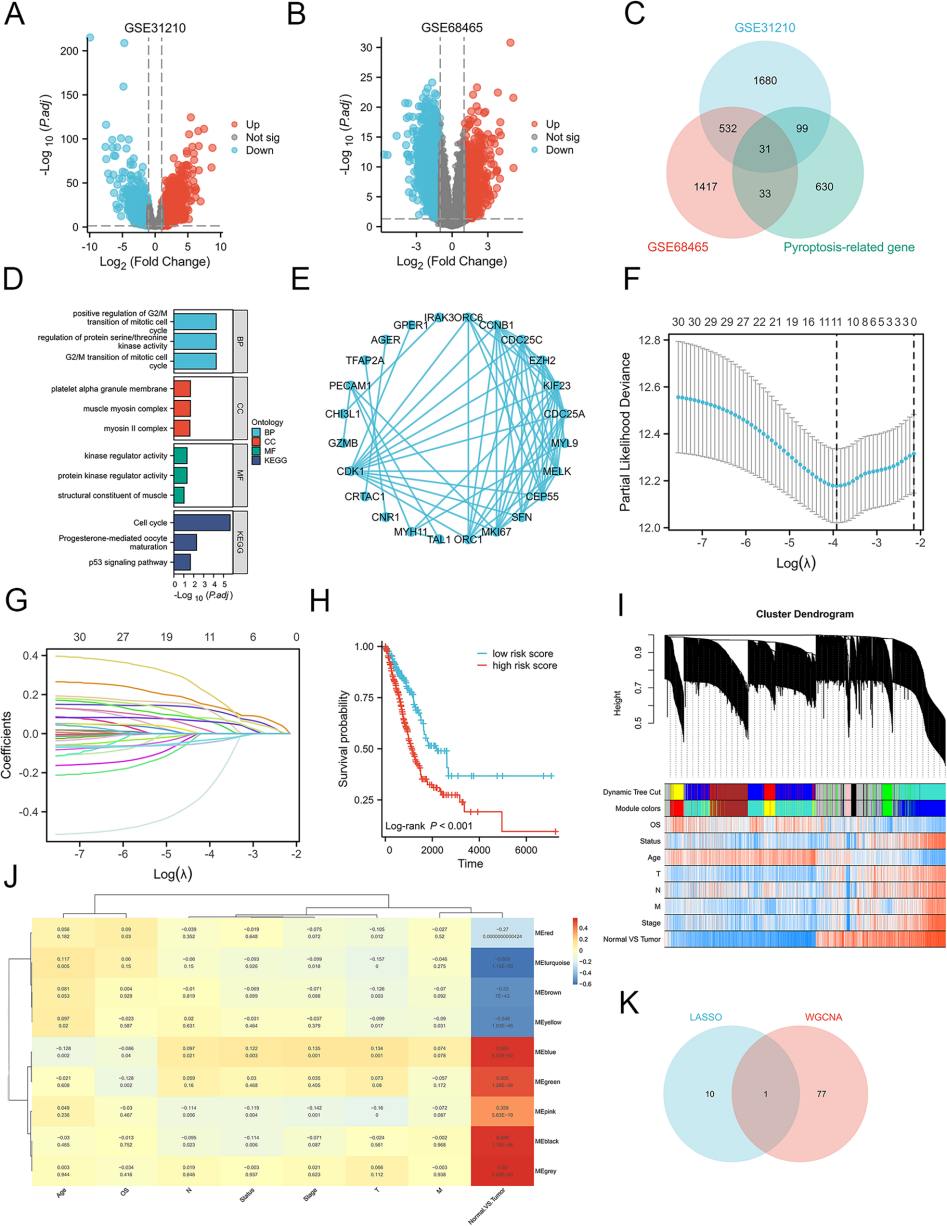
Identification of pyroptosis-related DEGs and the LASSO and WGCNA analysis in LUAD. **(A**,**B)** Volcano plot in GSE31210 and GSE68465. The red points represent upregulated genes screened on the basis of *p.adj* < 0.05 and |log_2_FC|> 1. The blue points represent downregulation of the expression of genes screened on the basis of *p.adj* < 0.05 and |log_2_FC|> 1. The gray points represent genes with no significant difference. **(C)** Overlap of DEGs and Pyroptosis-related genes. **(D)** The bubble chart of GO annotation and KEGG pathway enrichment analysis of pyroptosis-related DEGs. GO annotation divided pyroptosis-related DEGs into three functional groups: biological processes, cell composition, and molecular function. The size of the bubble indicates the number of genes, and the color of the bubble from red to green represents the significance of enrichment. **(E)** PPI network analysis of pyroptosis-related DEGs. **(F)** Cross-validation for tuning parameter selection in the LASSO regression. **(G)** Coefficient profiles in the LASSO regression. **(H)** Kaplan–Meier survival curves of high- and low- risk patients in TCGA-LUAD. **(I)** The cluster dendrogram of genes of LUAD patients. Each branch in the figure represents one gene, and every color below represents one co-expression module. **(G)** Correlation between the gene module and clinical characteristics. **(K)** Overlap of Pyroptosis-related DEGs and genes in the turquoise module of WGCNA. FC, fold change; DEGs, differentially expressed genes; GO, gene ontology; BP, biological processes; CC, cell composition; MF, molecular function; KEGG, Kyoto Encyclopedia of Genes and Genomes; PPI, protein–protein interaction. LASSO, least absolute shrinkage and selection operator; WGCNA, weighted genes co-expression network analysis; LUAD, lung adenocarcinoma.

As shown in Fig. 1, there is a flowchart for the analysis process. By conducting a differential analysis of the GSE31210 and GSE68465 datasets, it was observed that 1153 genes exhibited up-regulation and 1189 genes displayed down-regulation (*p.adj* < 0.05, |log_2_FC|> 1) from GSE31210, while 852 genes exhibited up-regulation while 1161 genes displayed down-regulation (*p.adj* < 0.05, |log_2_FC|> 1) from GSE68465 (Fig. 2A and B). Additionally, by entering the keyword “pyroptosis gene” in the GeneCard database, we obtained 792 pyroptosis-related protein coding genes (relevance score > 1). Subsequently, we identified 31 genes of interest by overlapping 2342 DEGs in GSE31210, 2013 DEGs in GSE68465, and 792 pyroptosis-related protein coding genes (Fig. 2C). Moreover, GO and KEGG analysis were performed on 31 pyroptosis-related genes. The result of GO indicate that 31 pyroptosis-related genes were primarily involved in protein kinase regulatory activity and kinase regulatory activity, among other functions. Additionally, following KEGG pathway analysis, it was determined that the cell cycle and p53 signaling pathway were the primary pathways enriched (Fig. 2D). In addition, a PPI network analysis of DEGs was also performed (Fig. 2E).

The LASSO regression algorithm was applied to refine a set of 31 pyroptosis-related genes. By calculating regression coefficients, we identified a signature consisting of 11 genes with the highest predictive value (Fig. 2F,G). Subsequently, survival analysis was performed, revealing that individuals belonging to the high-risk score group exhibited a significantly lower OS rate compared to those belonging to the low-risk score group (*p* < 0.05, Fig. 2H). These findings may suggest that when it comes to LUAD patients, a high-risk score may be a poor prognostic indicator. Rather than only focusing on DEGs, WGCNA method performs a comprehensive association analysis of genes and clinical phenotypes in addition to identifying gene sets of interest. By modifying the correlation between numerous genes and phenotypes, this methodology converts the task of adjusting for multiple hypothesis testing into the linkage of multiple gene sets. During the course of our research, we employed average linkage hierarchical clustering methodology to detect nine co-expression modules. Among these modules, the turquoise module, comprising 532 genes, exhibited the most robust correlation with disease (r = 0.61, *p* < 0.0001, Fig. 2I,J). Subsequently, we selected 78 genes from the turquoise module as hub genes (|MM|> 0.75 and |GS|> 0.5). Through the overlap of 11 pyroptosis-related DEGs and 78 disease-associated genes, we ultimately identified CRTAC1 as the hub gene (Fig. 2K). Given the lack of comprehensive research on the role of CRTAC1 in LUAD, we proceeded to investigate its effects and underlying mechanisms.

### CRTAC1 expression in LUAD and its prognostic and diagnostic value

**Figure 3:**
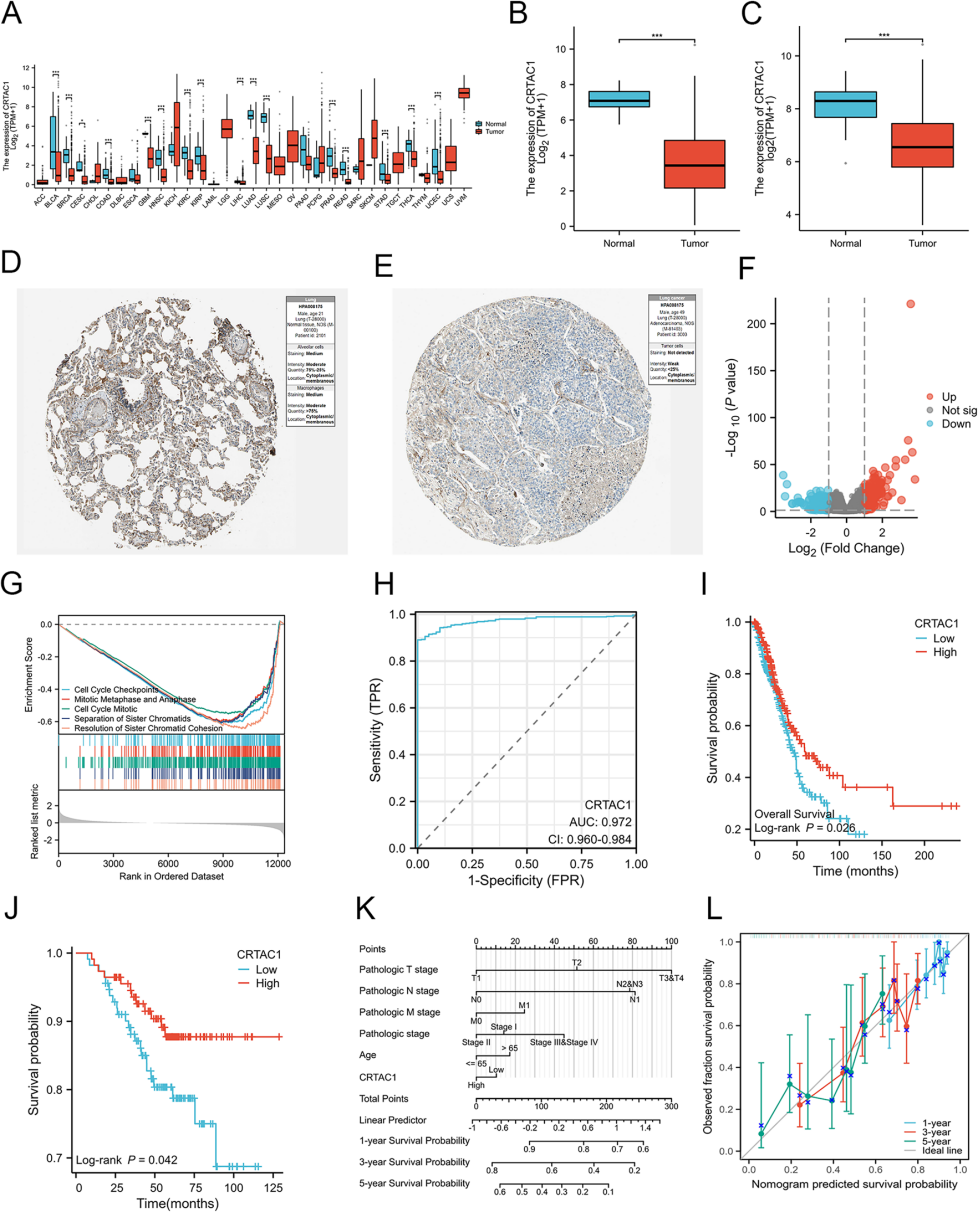
Analysis of the differential expression of CRTAC1 and its prognostic and diagnostic value in LUAD. **(A)** CRTAC1 mRNA expression was significantly reduced in the majority of tumors compared to normal tissues. **(B**,**C)** CRTAC1 mRNA expression was significantly lower in LUAD tissues as compared to normal tissues based on TCGA and GSE31210. **(D**,**E)** CRTAC1 protein expression was significantly lower in LUAD tissues compared to normal tissues based on Human Protein Atlas. **(F)** Volcano plot of DEGs between the low and high CRTAC1 expression groups. The red points represent upregulated genes screened on the basis of *p.adj* < 0.05 and |log_2_FC|> 1. The blue points represent downregulation of the expression of genes screened on the basis of *p.adj* < 0.05 and |log_2_FC|> 1. The gray points represent genes with no significant difference. **(G)** GSEA showed five pathways enriched in the low CRTAC1 groups. **(H)** The ROC curves demonstrated that CRTAC1 exhibited superior predictive efficacy (AUC = 0.972). **(I**,**J)** Kaplan‐Meier survival curves of LUAD patients from TCGA and GSE31210 showed the high CRTAC1 mRNA expression group exhibited a more favorable prognosis compared to the low expression group. **(K)** A nomogram integrated CRTAC1 expression and clinicopathological characteristics for predicting the probability of 1-, 3- and 5-year OS. **(L)** Calibration curve analysis results suggested that the predicted survival probabilities closely approximated the ideal line for 1-, 3-, and 5-year overall survival. ****p < 0.0001, ***p < 0.001, **p < 0.01, *p < 0.05, ns, no significance. DEGs, differentially expressed genes; FC, fold change; GSEA, Gene Set Enrichment Analysis; ROC, receiver operating characteristic curves; AUC, area under the ROC curve; OS, overall survival.

The mRNA expression levels of CRTAC1 were significantly decreased in LUAD and various other types of cancer (*p* < 0.05) (Fig. 3A,B,C). The HPA database’s Immunohistochemistry (IHC) results further confirmed the low CRTAC1 expression at protein level in LUAD (Fig. 3D,E). Furthermore, in the lung cancer cohort, lower CRTAC1 expression was in connection with advanced pathologic stage (*p* = 0.004), pathologic T stage (*p* = 0.006), and pathologic N stage (*p* = 0.03) (Table 1). Subsequently, using the median of CRTAC1 expression as the cut-off point, we categorized patients into groups with low and high expression and examined the DEGs within the TCGA-LUAD cohort. Among the 12,470 protein-coding genes, 181 genes exhibited up-regulation, and 189 genes displayed down-regulation (*p.adj* < 0.05, |log_2_FC|> 1) (Fig. 3F). GSEA showed that the low CRTAC1 expression group was significantly enriched in cell cycle checkpoints, cell cycle mitotic, and resolution of sister chromatid cohesion (Fig. 3G). Additionally, we examined the diagnostic and prognostic significance of CRTAC1 based on the TCGA-LUAD cohort. According to the result of ROC curve, the area under the curve (AUC) for CRTAC1 was 0.972, demonstrating its robust diagnostic value (Fig. 3H). Additionally, in comparison to patients exhibiting low CRTAC1 expression, those demonstrating high CRTAC1 expression have a better prognosis in TCGA-LUAD (*p* = 0.026) and in GSE31210 (*p* = 0.042) (Fig. 3I,J). In order to predict patient risk, a nomogram was developed that integrates the CRTAC1 expression level and clinicopathological characteristics (Fig. 3K). Subsequently, the calibration curve results demonstrated ideal congruity between the observed and predicted the observed and projected overall survival rates for 1, 3, and 5 years (Fig. 3L). Above all, the expression of CRTAC1 may hold significant diagnostic and prognostic implications for lung adenocarcinoma.

### The TF network and miRNA network

**Figure 4:**
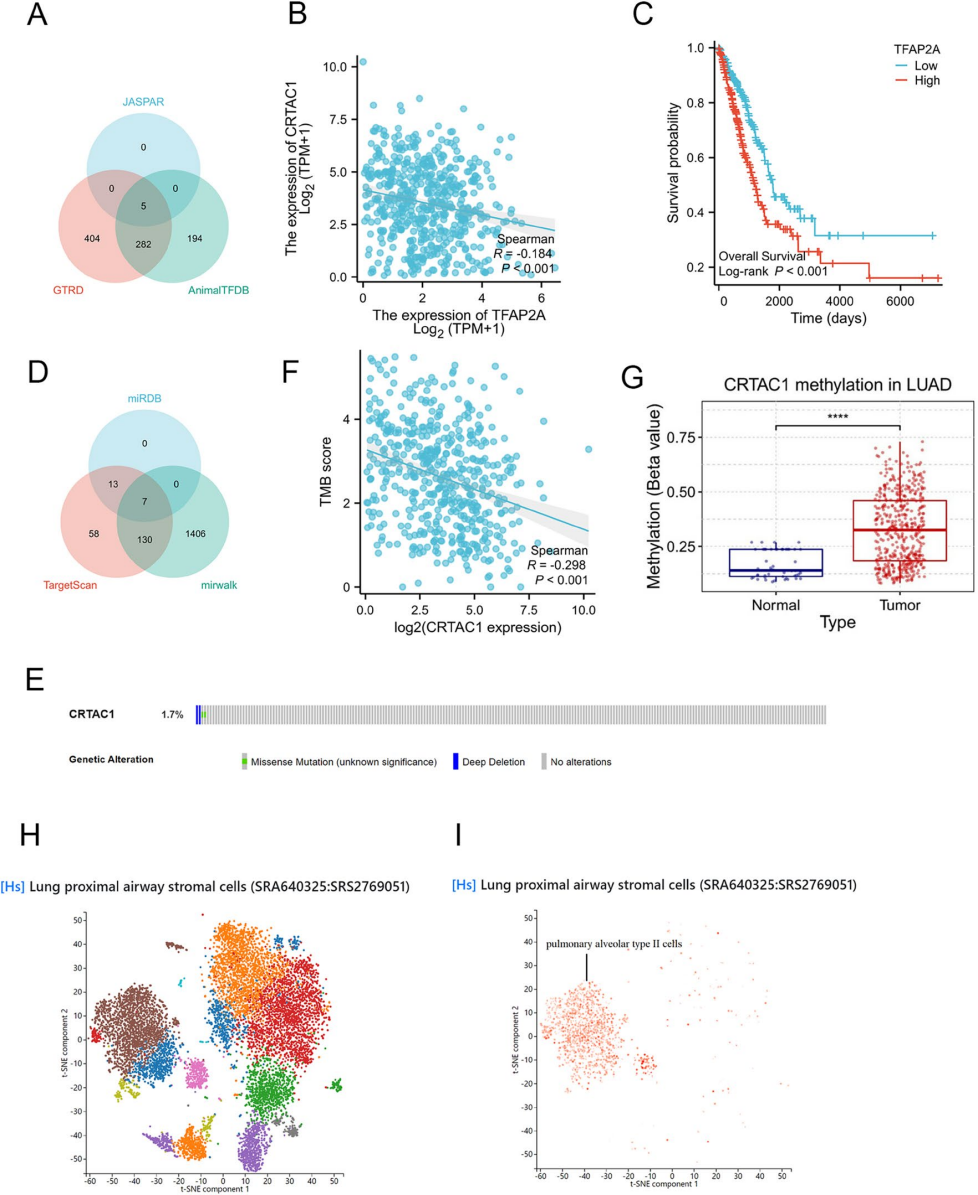
CRTAC1 upstream regulatory mechanism analysis, mutational analysis, methylation analysis and single-cell analysis. **(A)** By overlap of TFs based on JASPAR, GTRD, and AnimalTFDB, five TFs were ultimately identified. **(B)** Scatter plot showed a statistically significant negative correlation between the levels of CRTAC1 and TFAP2A expression. **(C)** Kaplan‐Meier survival curves of TCGA-LUAD patients showed the low TFAP2A mRNA expression group exhibited a more favorable prognosis compared to high expression group. **(D)** By overlap of miRNA based on miRDB, TargetScan, and mirwalk, seven miRNA were ultimately identified. **(E)** Frequency and type of CRTAC1 mutations in LUAD. **(F)** Scatter plot showed a statistically significant negative correlation between CRTAC1 expression and TMB in LUAD. **(G)** The methylation expression levels of CRTAC1 in LUAD tissues are significantly higher compared to those in normal tissues. **(H)** Single-cell RNA sequencing location analysis of CRTAC1. **(I)** CRTAC1 was enriched in pulmonary alveolar type II cell clusters. ****p < 0.0001, ***p < 0.001, **p < 0.01, *p < 0.05, ns, no significance. TF, transcription factor; TMB, tumor mutation burden.

To investigate the upstream regulatory mechanism of CRTAC1, our study examined the potential linkages between CRTAC1, TFs and miRNAs. A comprehensive search of the JASPAR, GTRD, and AnimalTFDB databases yielded 5, 691, and 481 TFs, respectively, that were potentially targeting CRTAC1. By intersecting the results from the three databases, TFAP2A, PAX5, AR, RUNX1, and NR2F1 were identified as the final candidates (Fig. 4A). Moreover, the levels of expression of the five potential TFs were analyzed, as well as their correlation with CRTAC1 in the TCGA-LUAD cohort (Supplement Fig. 1A). According to the findings, TFAP2A expression was up-regulated in tumor tissue and negatively correlated with CRTAC1 expression (r = − 0.184, *p* < 0.001) (Fig. 4B). Survival analysis revealed a more favorable prognosis for LUAD patients with low levels of TFAP2A (Fig. 4C). Next, utilizing miRWalk, miRDB, and TargetScan datasets, we conducted an investigation into the upstream miRNA regulator of CRTAC1. Through the integration of outcomes from the aforementioned datasets, we identified seven potentially significant miRNAs (Fig. 4D). Finally, we established the TF network and miRNA network (Supplement Fig. 1B,C), which may regulate the expression of CRTAC1.

### CRTAC1 mutational analysis, methylation analysis and single-cell analysis

The etiology of cancer involves the accumulation of somatic mutations and aberrant DNA methylation. Thus, our research employed cBioPortal to examine the alterations in CRTAC1 gene mutations in TCGA-LUAD. The outcome revealed the CRTAC1 mutation rate of merely 1.7%, indicating a high degree of conservation. Additionally, Missense Mutation and Deep Deletion were identified as the prevalent types of gene alteration (Fig. 4E). Then, we correlated the CRTAC1 expression levels with their corresponding mutation load and found a trend of negative correlation between gene expression and TMB (Fig. 4F). Besides, employing methylation analysis via the GSCA database, a significant increase in DNA methylation of CRTAC1 was found in LUAD tissue (*p* < 0.001) (Fig. 4G). Subsequently, CRTAC1 mRNA expression and its methylation levels exhibited a significant negative correlation in LUAD (r = − 0.24, *p* < 0.001) (Supplement Fig. 1D). Additionally, utilizing the PanglaoDB dataset, CRTAC1 was predominantly expression in pulmonary alveolar type II cell clusters in accordance with single-cell analysis (Fig. 4H,I). Based on the findings of prognostic analysis, it can be inferred that a decrease in CRTAC1 expression within pulmonary alveolar type II cells may contribute significantly to LUAD's development, as patients with low CRTAC1 expression exhibited a poor prognosis.

### Correlation analysis of CRTAC1 expression with immune cell infiltration and immune checkpoint genes

**Figure 5:**
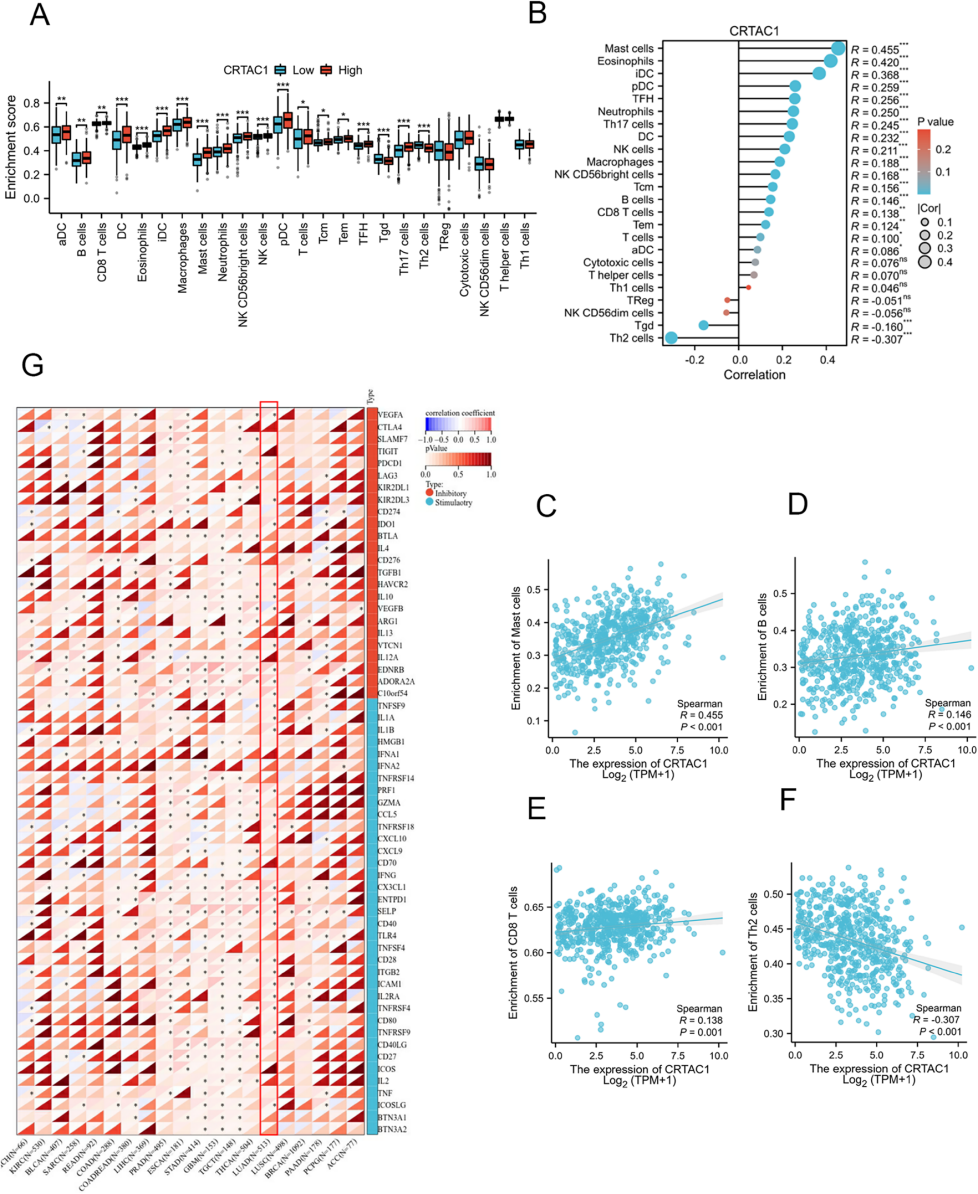
The correlation analysis of CRTAC1 with immune cell infiltration and immune checkpoint. **(A)** Comparisons of infiltration levels of immune cells between the low and high CRTAC1 expression groups with the ssGSEA algorithm. **(B)** The lollipop plot illustrated the correlation between CRTAC1 expressions and 24 different immune cells. **(C**,**D**,**E)** The scatter plot revealed a statistically significant positive correlation between CRTAC1 expression and Mast cells, B cells and CD8 T cells. **(F)** The scatter plot revealed a statistically significant negative correlation between CRTAC1 expression and Th2 cells. **(G)** Comparisons of the expression of immune checkpoints between the low and high CRTAC1 expression groups in LUAD and various other types of cancer. The red represents inhibitory genes. The blue represents stimulatory genes. ****p < 0.0001, ***p < 0.001, **p < 0.01, *p < 0.05, ns, no significance.

The present study conducted a correlation analysis between the CRTAC1 expression and the immune cell infiltration as well as the presence of immune checkpoint genes within tumor immune microenvironment. To this end, we conducted an analysis of the enrichment scores of 24 distinct immune cell types in two groups (Fig. 5A), and found a significant positive association between the CRTAC1 expression and most immune cell infiltration (Fig. 5B), such as Mast cells (r = 0.455, *p* < 0.001), B cells (r = 0.146, *p* < 0.001), and CD8 T cells (r = 0.138, *p* = 0.001) (Fig. 5C,D,E). There was, however, a negative correlation between CRTAC1 expression and Th2 cells (r = − 0.307, *p* < 0.001) (Fig. 5F). Additionally, our study indicated that CRTAC1 expression correlated positively with the majority of immune checkpoint genes in diverse cancer types (Fig. 5G). It is worth noting that our analysis of LUAD has identified significant differential expression in 27 out of 50 immune checkpoint genes, including 10 inhibitory genes (VEGFA, KIR2DL3, IDO1, HAVCR2, IL10, VEGFB, VTCN1, EDNRB, ADORA2A, and C1OorF54) and 17 stimulatory genes (TNFSF9, ILIA, ILIB, PRF1, CCL5, TNFRSF18, CXC3CL1, ENTPD1, SELP, CD40, CD28, ITGB2, ICAM1, IL2RA, TNFRSF4, TNFRSF9, and ICOSLG). The majority of the 27 genes demonstrated significantly higher expression levels in the high CRTAC1 expression group. These findings suggested that patients with high CRTAC1 expression may experience improved outcomes with immunotherapy.

### Prediction of drug sensitivity

According to the drug sensitivity analysis, 77 out of 198 drugs were significantly different between the low and high CRTAC1 expression groups in the GDCS. Notably, commonly used drugs in the treatment of lung cancer, such as Docetaxel, Cisplatin, Vinblastine, and Paclitaxel, were among those drugs (Supplement Fig. 2A,B,C,D). There was a higher level of drug response in patients with high CRTAC1 expression, suggesting a potentially favorable prognosis for this patient group.

### CRTAC1 expression levels in real-world cohorts validated

**Figure 6:**
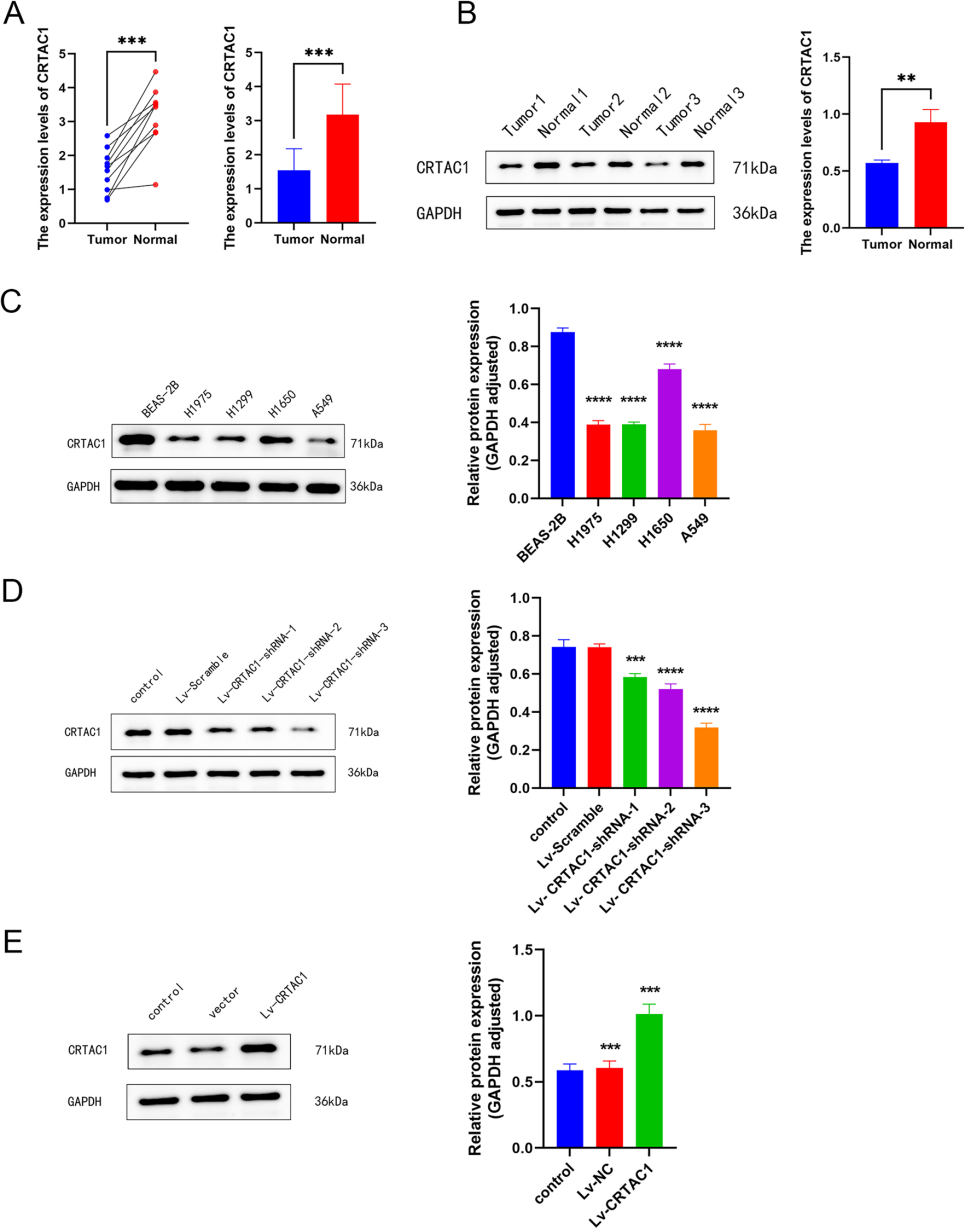
The mRNA and protein expression of CRTAC1. **(A)** qPCR detected the tumor tissues had significantly lower mRNA expression levels of CRTAC1 than the adjacent normal tissues. **(B)** WB detected the tumor tissues had significantly lower protein expression levels of CRTAC1 than the adjacent normal tissues. **(C)** WB detected the protein expression levels of CRTAC1 in different LUAD cell lines. **(D**,**E)** WB was used to assess the interference and overexpression efficiency of CRTAC1 in H1650 and A549 cell lines. ****p < 0.0001, ***p < 0.001, **p < 0.01, *p < 0.05, ns, no significance.

Acknowledging the potential for inaccuracies in our bioinformatics analysis, we conducted a series of validation experiments to substantiate the credibility of our results. At the outset, surgical resection samples of tumor and adjacent normal tissues were obtained from 10 patients diagnosed with LUAD at Tianjin Chest Hospital for the qPCR. According to the outcomes of qPCR, the adjacent normal tissue had significantly higher mRNA levels of CRTAC1 than the tumor tissue (Fig. 6A). Furthermore, 3 patient samples were randomly selected from a total of 10 for the WB. The result of WB confirmed the augmented protein expression of CRTAC1 in the adjacent normal tissue relative to the tumor tissue (Fig. 6B).

### Verification experiments for bioinformatics analysis

**Figure 7:**
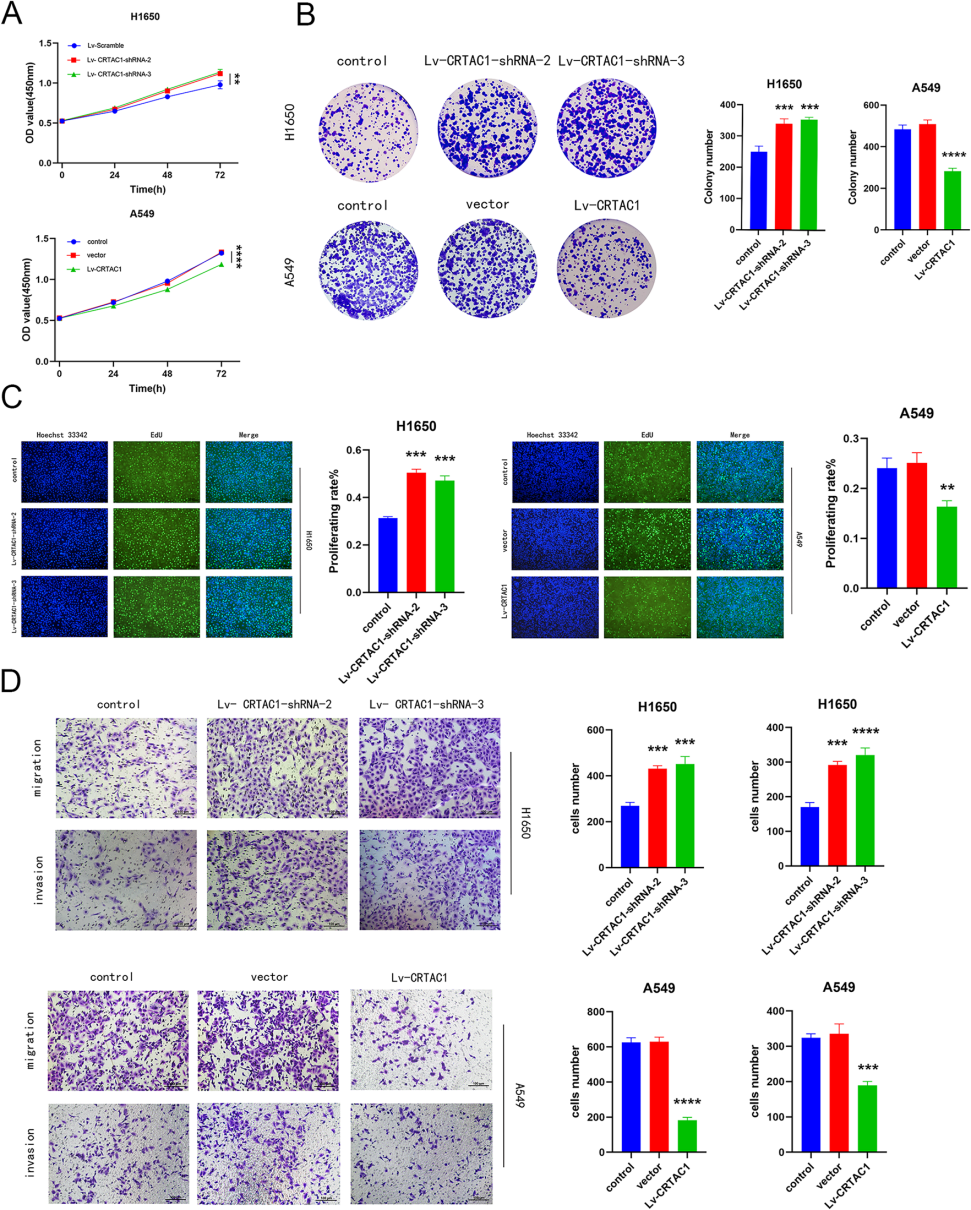
Cellular experiments. **(A)** CCK8 assay showing the impact of CRTAC1 expression level on the proliferative capabilities of H1650 and A549 cell lines. **(B)** Colony formation assay assessing the effect of CRTAC1 on cell proliferation. **(C)** EdU assay evaluating cellular proliferative activity. **(D)** Transwell assay verifying the impact of CRTAC1 on cell migration and invasion. ****p < 0.0001, ***p < 0.001, **p < 0.01, *p < 0.05, ns, no significance.

The results of the WB analysis demonstrated a statistically significant reduction in the expression levels of CRTAC1 in four LUAD cell lines (A549, H1650, H1975, H1299) when compared to the normal lung epithelial cell line (BEAS-2B) (Fig. 6C). Among the four LUAD cell lines, CRTAC1 exhibited relatively high expression in H1650 cell line, moderate expression in H1650 and H1299 cell lines, and low expression in A549 cell line. Consequently, we opted to employ the high-expression H1650 cell line and the low-expression A549 cell line for interference and overexpression, respectively, in order to carry out subsequent cell biology experiments. Lentiviral shRNA vectors and lentiviral expression vectors were employed to induce interference and overexpression of CRTAC1 expression in H650 cells and A549 cells, respectively. The expression efficiency was subsequently confirmed through WB technique (Fig. 6D,E). CCK8 assay showed that cell viability was significantly increased after CRTAC1 interference, while it was significantly decreased after CRTAC1 overexpression (Fig. 7A). Additional colony formation experiments provided further evidence that the downregulation of CRTAC1 expression in H1650 cell lines led to an increased formation of colonies. Conversely, the upregulation of CRTAC1 in A549 cell lines produced the opposite outcome (Fig. 7B). The findings from the EdU experiment provided additional evidence supporting the involvement of CRTAC1 in the suppression of proliferation in LUAD cell lines, as indicated by the decreased rates of proliferation observed in cells overexpressing CRTAC1 (Fig. 7C). Lastly, the results derived from migration and invasion experiments demonstrated a noteworthy decrease in the migratory and invasive potential of lung adenocarcinoma cells following the overexpression of CRTAC1 (Fig. 7D). These findings suggest that the modulation of CRTAC1 could potentially serve as an innovative therapeutic approach for the treatment of LUAD.

## Discussion

In 2015, pyroptosis was defined as a sort of programmed cell death that is mediated by gasdermin^13^. For the past few years, a substantial body of evidence has emerged linking pyroptosis to a variety of pathologies including nervous system diseases, infectious diseases, cardiovascular diseases, autoimmune disorders, and neoplastic growths^14–17^. Within the domain of lung cancer, pyroptosis has also been shown to contribute to tumor development and occurrence, tumor immune microenvironment (TIME), and immunotherapy responses^7,18,19^. In our study, by intersecting the identified DEGs with pyroptosis-related genes, a total of 31 pyroptosis-related DEGs were identified. Subsequent KEGG pathway analysis of these genes indicated significant enrichment in the Cell cycle signaling pathways. The cell cycle machinery plays a crucial role in the regulation of developmental genes and the determination of cell fate^20^. Research has demonstrated that pyroptosis may regulate the expression of cell cycle-related proteins, affect the progression of cells from S to G2 phase, and consequently influence the proliferation of tumor cells^21^. Following LASSO analysis, 11 hub genes were identified and a prognostic signature associated with pyroptosis was established. Subsequently, patients were categorized into low- and high-risk score groups based on the median risk score. Patients with a high-risk score demonstrated a poor prognosis, consistent with previous studies.^6,22^. Finally, after overlapping the hub genes from LASSO and WGCNA analysis, CRTAC1 was determined to be one of the most significant pyroptosis-related genes. Furthermore, our investigation explored the alterations in CRTAC1 expression and mutation during the malignant progression of LUAD, as well as the effects of CRTAC1 on both the immune cell infiltration and immune checkpoint genes.

As a glycosylated extracellular matrix, CRTAC1 is secreted by chondrocytes in the articular cartilage^23^. Several prior studies have demonstrated that patients with decreased CRTAC1 expression exhibit an unfavorable prognosis in glioma, bladder cancer, and gastric adenocarcinoma^9,10,24^. Jia et al. reported that elevated CRTAC1 expression is linked to earlier T (T1-2) stage, N (N0) stage, earlier pathologic stage (I and II), and lower grade in bladder cancer patients, suggesting that CRTAC1 may serve as a suppressor gene^25^. Our investigation similarly revealed a notable association between CRTAC1 expression and LUAD prognosis, with higher expression levels of CRTAC1 indicating a more favorable prognosis. Additionally, our analysis also found that elevated CRTAC1 expression was significantly associated with earlier pathologic T stage (*p* = 0.006), earlier pathologic N stage (*p* = 0.03), and earlier pathologic stage (*p* = 0.004), indicating that the CRTAC1 gene serves as a protective factor in the context of lung cancer.

Moreover, through the utilization of GSEA analysis, it was determined that the low CRTAC1 expression group exhibited a statistically significant enrichment in cell cycle checkpoints, mitotic metaphase and anaphase, and cell cycle mitotic pathways. These pathways are recognized to exert pivotal functions in cancer progression. For example, defects in cell cycle checkpoints could increase the risk of lung cancer^26^. Furthermore, errors during cell division have also been observed to cause aberrations in chromosome content and disturb the equilibrium of chromosome numbers, thereby facilitating the onset of cancer^27^. These may contribute to poor prognosis in patients with low CRTAC1 expression.

In addition, our research explored the upstream regulatory mechanism of CRTAC1 through the screening of its TFs and miRNA. We found that TFAP2A is a key transcription factor for CRTAC1 and high levels of TFAP2A in LUAD patients are linked to a poorer prognosis, in alignment with prior research^28,29^. Additionally, we discovered a negative relationship between TFAP2A and CRTAC1 expression, indicating that increased TFAP2A levels may decrease CRTAC1 expression and contribute to disease progression in LUAD patients. Research has demonstrated a significant association between miRNAs and the development of lung cancer, particularly NSCLC^30^. MiRNAs modulate gene expression by impacting the translational activity of their specific mRNA targets, and play a role in various biological processes including cell differentiation, proliferation, and apoptosis^31,32^. Our research identified seven miRNAs that are potentially involved in the regulation of CRTAC1 expression.

The role of gene mutations in progression of LUAD is noteworthy, and the detection of such mutations holds potential for the exploration of targeted therapeutic interventions in the management of lung cancer. Shen's research indicated that the CRTAC1 mutation rate in gastric adenocarcinoma is a mere 2%, with missense mutation being the most prevalent form of gene alteration^24^. Our analysis of the cBioPortal dataset confirmed that the CRTAC1 mutation rate in LUAD is only 1.7%, with missense mutation and deep deletion being the most common types of gene alteration. Additionally, as CRTAC1 expression decreased, there was a corresponding increase in tumor mutation load. These findings indicate that CRTAC1 has a low mutation rate and functions as a stable tumor suppressor gene. Modulating the expression of CRTAC1 may effectively decrease tumor mutation load, suggesting that CRTAC1 could serve as a promising target for targeted therapy in LUAD. Studies have shown that DNA hypermethylation can provide supplementary information for the diagnosis and prognosis of cancer^33–35^. Our research revealed a significant elevation in the DNA methylation level of CRTAC1 in LUAD tissue relative to normal tissue, suggesting the potential role of CRTAC1 in cancer suppression. It has been reported that lung adenocarcinoma cells originate from AT2 epithelial cells^17,36^. Our analysis at the single-cell level has revealed that CRTAC1 is predominantly expressed in alveolar AT2 cells and minimally expressed in tumor cells, indicating that decreased CRTAC1 expression may be a significant factor in the oncogenic progression of epithelial cells.

TIME consists of non-tumor elements, including immune cells and cytokines, with immune cells playing a crucial role in the tumor immune micro environment^37^. Research has demonstrated a correlation between increased immune cell infiltration and a more favorable prognosis in patients with lung adenocarcinoma^38^. Our research has unveiled a positive association between CRTAC1 expression and the levels of infiltration of various immune cell types. Further analysis showed that there was a noteworthy rise in the proportions of Mast cells, B cells, and CD8 T cells in the high CRTAC1 expression group, as compared to the low CRTAC1 expression group.

Mast cells play a crucial role as immunoregulatory sentinel cells within the tumor microenvironment. Some researches demonstrated the enhanced abundance of Mast cells is associated with improved patient prognosis^39,40^. B cells are a crucial constituent of the tumor immune microenvironment, present throughout all stages of cancer, and exert significant influence on tumor development via antigen provision and cytokine secretion^41,42^. An increased infiltration of B cells serves as a positive prognostic factor and may enhance the efficacy of immunotherapy^43–45^. In addition, CD8 T cells are recognized as the preferred immune cells for targeting cancer and also serve as an essential parameter for the antitumor effect of immune checkpoint inhibitors^46,47^. Our research revealed that the upregulation of CRTAC1 is linked to improved disease progression, plausibly attributable to the augmented infiltration of different immune cells, including Mast cells, B cells and CD8 T cells.

The effectiveness of immunotherapy is reliant on the abundance of immune cell, as well as the sufficient expression of immune checkpoint genes. In our examination of immune checkpoint gene expression, 27 immune checkpoint genes were identified, which demonstrated a positive correlation with CRTAC1. This suggests that treatment with immune checkpoint inhibitors may yield greater efficacy in individuals with high CRTAC1 expression. Moreover, while targeted drugs and immune checkpoint inhibitors are gaining significance in the treatment of NSCLC, chemotherapy remains the primary therapeutic modality for lung NSCLC^48^. Jin’s research indicated that the potential of CRTAC1 to augment the chemosensitivity of non-small cell lung cancer to cisplatin is attributed to its ability to induce RyR-mediated calcium release and suppress Akt1 expression^12^. Therefore, we conducted an analysis of chemotherapeutic drugs and found that patients with higher levels of CRTAC1 expression showed increased sensitivity compared to those with lower levels of CRTAC1 expression.

The dysregulation of tumor cell proliferation represents a pivotal factor in the initiation and progression of neoplastic growth. Detection of cell proliferation generally involves analyzing changes in the number of cells during division, which reflects the growth state and activity of cells. Our research examined the impact of regulating CRTAC1 expression levels on the proliferation of lung adenocarcinoma cells. The findings indicated that upregulation of CRTAC1 led to a decrease in cell proliferation, thereby demonstrating the tumor-suppressive properties of CRTAC1. Tumor metastasis and invasion represent pivotal biological traits of malignant tumors. The Transwell assay, alternatively referred to as Transwell migration or invasion assays, is a widely employed method for evaluating the migratory and invasive capabilities of cells^49^. Migration assays are used to evaluate the capacity of cells to move on a two-dimensional plane, thereby providing insights into the dissemination of tumors from the primary location to adjacent tissues and serving as a preliminary indication of metastasis^50^. Invasion assays, on the other hand, enable the assessment of cell invasion capabilities by replicating the process of traversing the extracellular matrix, which is a crucial step for tumor cells to breach the basement membrane, gain access to the vascular or lymphatic systems, and ultimately metastasize to distant organs^51^. Our study suggested that overexpression of CRTAC1 can effectively inhibit the invasion and migration of lung adenocarcinoma cells. These findings have the potential to offer novel insights for the future treatment of LUAD.

Despite the numerous intriguing discoveries in our research, there exist certain constraints in this investigation. Additional in vitro and in vivo investigations are imperative to explicate and enhance our comprehension of the exact upstream and downstream mechanisms underlying CRTAC1.

## Conclusion

CRTAC1 exhibits the potential to function as both a diagnostic and prognostic biomarker, and it may hold significant implications for tumor immune microenvironment and drug therapy in LUAD.

### Supplementary Information

Supplementary Information.

## Data Availability

The data for this study were sourced from online databases, the details of which are stated in the article. For further inquiries, please contact the corresponding author.
