# Supplementary material for: CRTAC1 identified as a promising diagnosis and prognostic biomarker in lung adenocarcinoma
Source: Sci Rep. 2024 May 16;14:11223. doi: 10.1038/s41598-024-61804-x (PMC11099150; doi:10.1038/s41598-024-61804-x)
Supplement: Supplementary file 1 — Supplementary Information. [file 41598_2024_61804_MOESM1_ESM.pdf]

Supplement table 1: The primers of GAPDH and CRTAC1.

Primer name	Forward primers 5’ 3’
GAPDH-F	GAGAAGGCTGGGGCTCATTT
GAPDH-R	AGTGATGGCATGGACTGTGG
CRTAC1-F	CCCTGGCTGACTTCAACCGT
CRTAC1-R	ATGGAGAACTTGGGTGAGGC

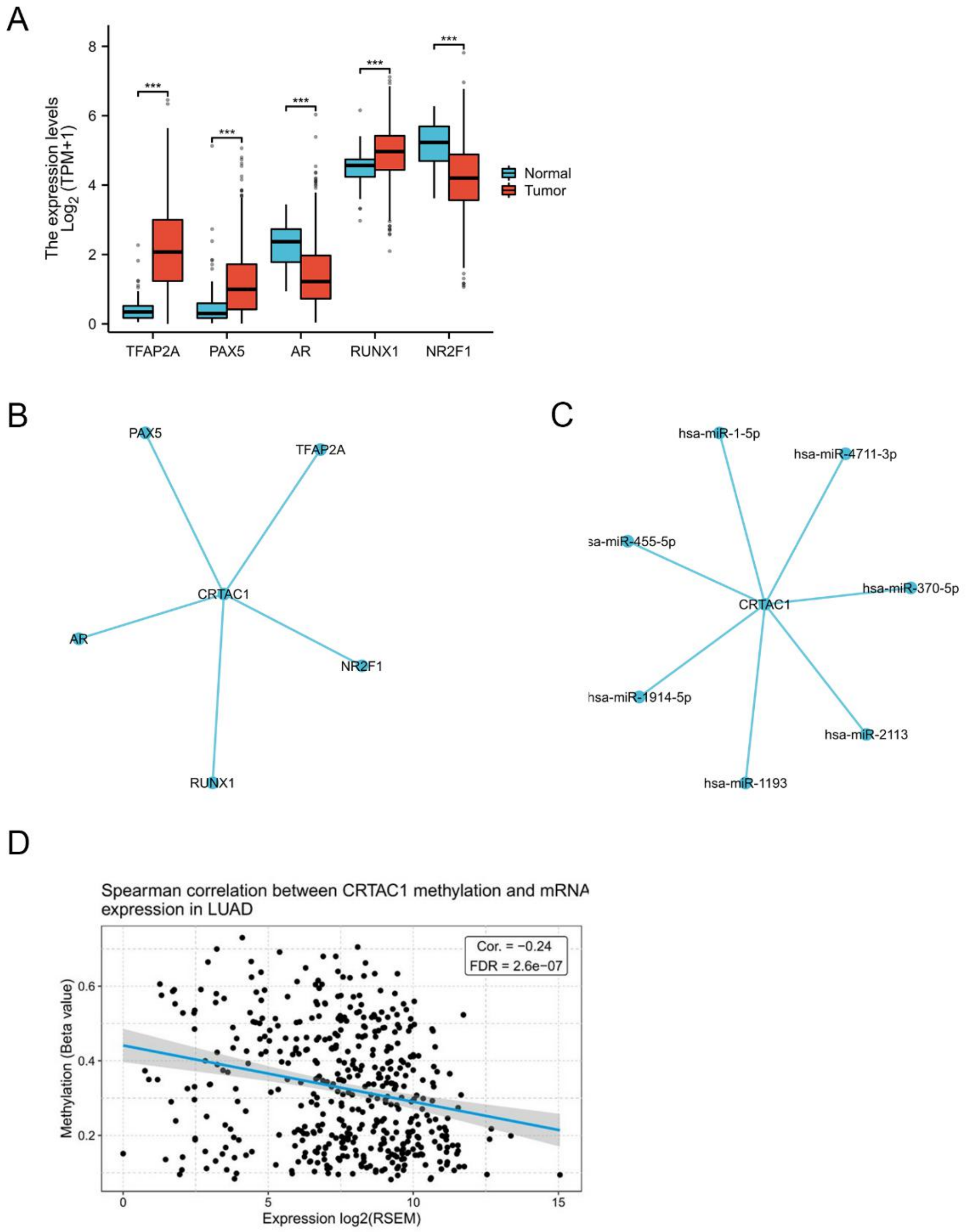

Supplement Figures 1: CRTAC1 upstream regulatory mechanism analysis and methylation analysis.

(A) The comparison of the differential expression levels of TFAP2A, PAX5, AR, RUNX1, and NR2F1 between normal and tumor tissue samples in TCGA-LUAD.

(B, C) The RFs and miRNA network of CRTAC1.

(D) Scatter plot showed a statistically significant negative correlation between CRTAC1 methylation and mRNA expression in LUAD.

Notes: ****p < 0.0001, ***p< 0.001, **p < 0.01, *p < 0.05, ns, no significance.

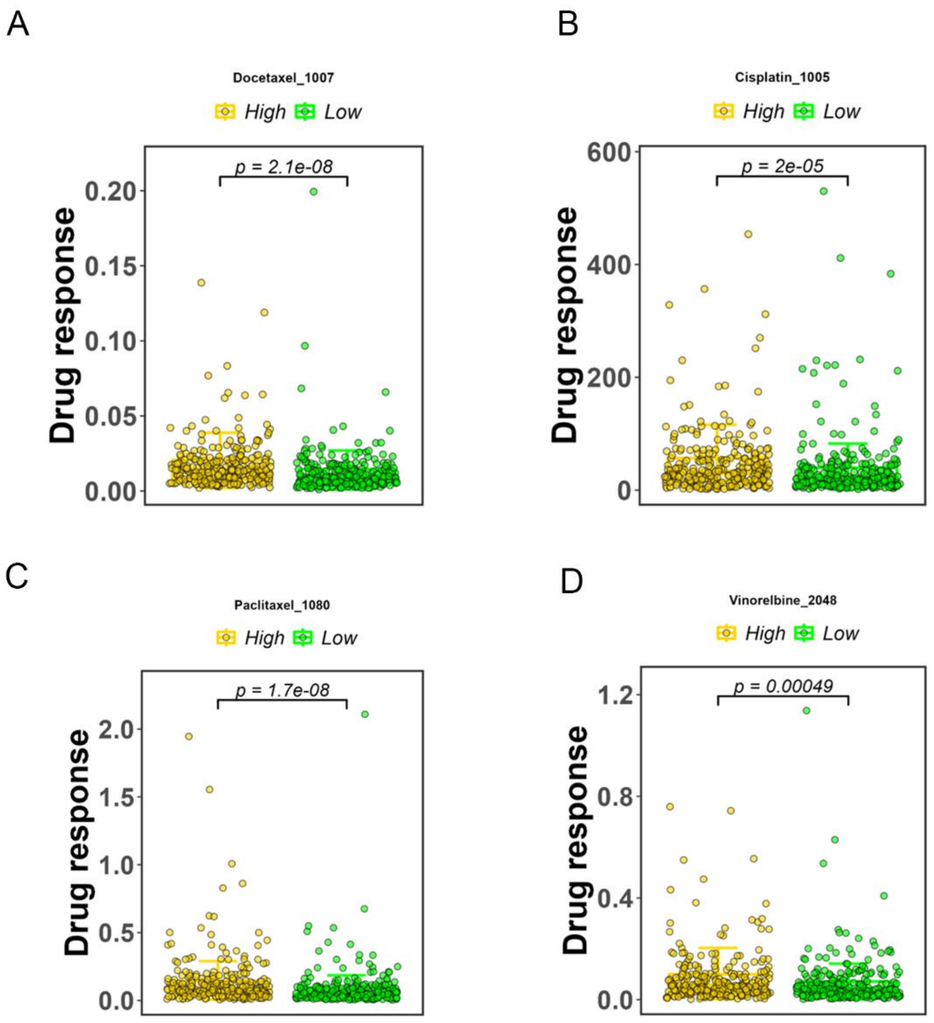

Supplement Figures 2: CRTAC1 drug sensitivity analysis.

(A, B, C, D) The high CRTAC1 expression group exhibited a greater sensitivity to Docetaxel, Cisplatin, Paclitaxel, and Vinblastine in comparison to low CRTAC1 expression group by GDSC database.
